# Opposite Effects of mGluR1a and mGluR5 Activation on Nucleus Accumbens Medium Spiny Neuron Dendritic Spine Density

**DOI:** 10.1371/journal.pone.0162755

**Published:** 2016-09-12

**Authors:** Kellie S. Gross, Dieter D. Brandner, Luis A. Martinez, M. Foster Olive, Robert L. Meisel, Paul G. Mermelstein

**Affiliations:** 1 Department of Neuroscience, University of Minnesota, Minneapolis, MN 55455, United States of America; 2 Graduate Program in Neuroscience, University of Minnesota, Minneapolis, MN 55455, United States of America; 3 Medical Scientist Training Program, University of Minnesota, Minneapolis, MN 55455, United States of America; 4 Department of Psychology, Arizona State University, Tempe, AZ 85287, United States of America; University of Texas at Austin, UNITED STATES

## Abstract

The group I metabotropic glutamate receptors (mGluR1a and mGluR5) are important modulators of neuronal structure and function. Although these receptors share common signaling pathways, they are capable of having distinct effects on cellular plasticity. We investigated the individual effects of mGluR1a or mGluR5 activation on dendritic spine density in medium spiny neurons in the nucleus accumbens (NAc), which has become relevant with the potential use of group I mGluR based therapeutics in the treatment of drug addiction. We found that systemic administration of mGluR subtype-specific positive allosteric modulators had opposite effects on dendritic spine densities. Specifically, mGluR5 positive modulation decreased dendritic spine densities in the NAc shell and core, but was without effect in the dorsal striatum, whereas increased spine densities in the NAc were observed with mGluR1a positive modulation. Additionally, direct activation of mGluR5 via CHPG administration into the NAc also decreased the density of dendritic spines. These data provide insight on the ability of group I mGluRs to induce structural plasticity in the NAc and demonstrate that the group I mGluRs are capable of producing not just distinct, but opposing, effects on dendritic spine density.

## Introduction

The group I metabotropic glutamate receptors (mGluRs), mGluR1a and mGluR5, are important modulators of neural signaling and plasticity. Located primarily on post-synaptic structures, they couple predominately to Gq G-proteins to activate a number of downstream signaling pathways that have both acute and long-term effects on neuronal excitability [1]. Given the variety of effects these receptors can have on neural plasticity and their wide expression throughout the brain, group I mGluRs are involved in virtually all nervous system functions and play important roles in a number of disease states, including drug addiction [2].

One way group I mGluRs may influence function is by regulating neural structure. In hippocampal neurons, for example, activating group I mGluRs with the pan-specific orthosteric agonist (*S*)-3,5-Dihydroxyphenylglycine (DHPG) leads to elongation of dendritic spines and decreases in spine density, suggesting a weakening and loss of synaptic connections [3–5]. Correlated with these structural changes, DHPG induces functional plasticity, leading to the development of long-term depression in both hippocampal pyramidal neurons and many other cell types [2]. This effect of group I mGluR activation on dendritic spine plasticity may have an important biological role as a mechanism of the normal activity-dependent synaptic remodeling and refinement that occurs as a result of learning or other experiences [5]. Additionally, group I mGluR regulation of spine structure has been linked to certain disease states. As examples, dysregulated mGluR5 signaling underlies spine abnormalities in models of Fragile X syndrome [6], and spine loss seen in models of Alzheimer’s disease [7].

Although the two group I mGluRs are closely related and couple to many of the same downstream signaling pathways, accumulating evidence demonstrates that mGluR1a and mGluR5 can also have distinct functions [8]. This observation highlights the importance of separately evaluating the influences of mGluR1a versus mGluR5, especially in regions where they are co-expressed, on neuronal function. Until now, the majority of studies looking at the effects of group I mGluRs on structural plasticity have not explored potential differences between these receptor subtypes. However, recent work examining estrogen effects on dendritic spine densities in the female nucleus accumbens (NAc) have provided *indirect* evidence of opposing functions of mGluR1a and mGluR5 in regulating structural changes. In the NAc core (NAcC), estradiol decreases spine density through an mGluR5 dependent mechanism [9]. Conversely, in the NAc shell (NAcSh), estradiol increases spine density via mGluR1a, an increase similar to what has been observed in the hippocampus and hypothalamus [9–11]. The possibility that the two group I mGluRs are having opposing effects on structural plasticity in the NAc is particularly intriguing as group I mGluR signaling in this region is thought to be heavily involved in the pathophysiology of addiction, and therapeutics are being developed for addiction that target the mGluR system [12].

With only ancillary evidence that mGluR1a and mGluR5 can have opposing effects on dendritic spine densities in the NAc, we sought to determine the effects of independent activation of these individual receptors on both spine density and morphology. Experiments were performed in ovariectomized female rats to eliminate estrogen as a source of activation of group I mGluR signaling [9]. Because of the high specificity and therapeutic potential of mGluR positive allosteric modulators (PAMs), we first looked at the effects of systemic treatment with an mGluR5 or an mGluR1 PAM. Direct infusion of a specific mGluR5 agonist into the NAc was also performed to determine whether local activation of mGluRs was sufficient to alter dendritic spines.

## Methods

### Animals

Female Sprague-Dawley rats (175–200 g) were ovariectomized by Envigo Laboratories (Indianapolis, IN) and arrived fully recovered from the procedure and in good health. Animals were pair housed and kept under a 14:10 light-dark cycle with lights on at 6 a.m. Food and water were available *ad libitum*. Animals were allowed to habituate for five days prior to experimentation. All animal procedures were in accordance with the National Institutes of Health Guidelines for the Care and Use of Laboratory Animals and were approved by the Animal Care and Use Committee at the University of Minnesota (Animal Welfare Assurance Number A3456-01).

### Drugs

3-Cyano-*N*-(1,3-diphenyl-1*H*-pyrazol-5-yl)benzamide (CDPPB) and (*RS*)-2-Chloro-5-hydroxyphenylglycine sodium salt (CHPG) were obtained from Tocris Bioscience (Minneapolis, MN). CDPPB was suspended in 0.5% methyl cellulose (w/v in deionized water, Sigma-Aldrich, St. Louis, MO). CHPG was dissolved in sterile saline. 9H-Xanthene-9-carboxylic acid (4-trifluoromethyl-oxazol-2-yl)-amide (SYN119) was synthesized by EAG Laboratories (Sunnyvale, CA) and was suspended in 20% 2-hydroxypropyl-β-cyclodextrin (w/v in deionized water, Sigma-Aldrich). For systemic treatments, CDPPB (5–10 mg/kg), SYN119 (10 mg/kg), or their corresponding vehicles were administered intraperitoneally (i.p.) at a volume of 2 ml/kg. For site-specific drug administration, CHPG (10 μg/0.5 μl/side) or an equivalent volume of vehicle was infused directly into the NAc. For all experiments, animals were sacrificed 24 hours after drug administration and tissue was processed for dendritic spine analysis.

### Surgical microinjection procedure

Animals were anesthetized with a 2.5–4% isoflurane (Piramal Critical Care, Bethlehem, PA)/oxygen mixture and placed in a stereotaxic apparatus. Drug or vehicle was injected bilaterally via a Hamilton microinjection syringe at the following coordinates targeting the core-shell border: AP: +1.80 mm from bregma, ML: ±1.50 mm from bregma, DV: -6.20 mm from dura. Infusions of 0.5 μl were given manually over the course of 2.5 minutes. The injection needle was then left in place for an additional 2.5 minutes to allow for diffusion of the drug away from the needle tip. Animals were given a subcutaneous injection of 2.5 mg/kg ketoprofen 20 minutes prior to surgery and every 12 hours after surgery to induce analgesia and a subcutaneous injection of 10 mg/kg Baytril at the time of surgery to prevent infection. Immediately following the surgery, animals were monitored until they recovered ambulatory posture. Afterwards, animals were observed for eating, drinking, and or the display of any discomfort. None of the animals exhibited any symptoms of problems. Location of the injection site was verified following euthanasia.

### Tissue preparation

Tissue was prepared and ballistically labeled with DiI following protocols previously established in our lab [9,13]. Twenty-four hours after drug treatment, animals were overdosed with Beuthanasia-D (0.3 ml i.p.; Schering, Union, NJ) and transcardially perfused with 25 mM phosphate buffered saline (PBS, pH = 7.2) for 3 minutes followed by 1.5% paraformaldehyde in PBS for 20 minutes. Brains were removed, coronally blocked, and then post-fixed in 1.5% paraformaldehyde for 1 hour before being stored in PBS. Brains were sliced into 200–300 μm sections through the striatum using a Vibratome (Leica VT1000 S, Buffalo Grove, IL). Sections were stored in PBS until DiI labeling.

### DiI labeling

DiI “bullets” were made by dissolving 2 mg DiI (Molecular Probes, Carlsbad, CA) in 100 μl dichloromethane and applying the solution to 90 mg of 1.3 μm tungsten particles (Bio-Rad, Hercules, CA). Particles were suspended in 10 ml of 15 mg/ml polyvinylpyrrolidone (PVP) and sonicated for 10 minutes with intermittent vortexing. The suspension was quickly pulled through a length of Teftzel tubing (Bio-Rad) pre-treated with PVP and allowed to settle before the remaining PVP was expelled. The tubing was dried for 20 minutes with nitrogen gas flow and then cut into 1.3 cm bullets. To deliver the DiI-coated tungsten particles, bullets were loaded into a Helios Gene Gun (Bio-Rad) with a modified barrel, 40 mm spacer, and 70 μm mesh filter. PBS was removed from wells containing brain sections, and one bullet was shot per section using helium gas at 100 PSI. After shooting, sections were stored overnight in the dark in PBS and then post-fixed in 4% paraformaldehyde for 1 hour the next day. Sections were mounted on slides and coverslipped with FluorGlo mounting media for lipophilic dyes (Spectra Services, Ontario, NY).

### Confocal imaging

Cells were imaged with a Leica TCS SPE confocal microscope (Leica, Manheim, Germany). All images were taken at a *xy* pixel distribution of 512 x 512 and a frequency of 400 Hz. Whole medium spiny neurons were imaged at 20X magnification with a z-step size of 1 μm and reconstructed using the Leica LAS AF software to measure the distance from the soma to each dendritic segment. Cells were imaged from the NAcC and NAcSh in all experiments, and cells were also imaged in the dorsolateral caudate for experiments using systemic drug manipulations. Data from a minimum of 7 animals per treatment group were collected for each experiment (see figure legends for specific group sample sizes). For each animal, each brain region had a minimum of six dendritic segments imaged for analysis, with two or three distal dendritic segments (distance of 70–200 μm from the soma) taken from each of two or three cells per brain region. Distal dendritic segments were chosen for analysis as this dendritic region of medium spiny neurons receives the majority of inputs from glutamatergic projections to the NAc [14]. Images of individual dendrites were taken with a 63X oil immersion lens and 5.6 optical zoom with a z-step size of 0.12 μm.

### Quantitation

Confocal images were processed through 3D deconvolution using Autoquant X3 AutoDeblur software (Media Cybernetics, Bethesda, MD) and reconstructed z-stacks were rendered by the Surpass module of Imaris software (Bitplane, Inc., St. Paul, Minnesota). Dendrites were manually traced in the *xy* plane using the Filament tool and Autodepth function by an investigator blinded to treatment condition. An accurate reconstruction of the diameter of the dendritic shaft, spine necks, and spine heads was rendered using the diameter function with a contrast threshold of 0.7. Spine density was calculated by summing the number of spines for the total dendrite length and then calculating the average spine density/10 μm. Densities were averaged across each cell and then within each brain region for each animal, providing a region-specific spine density average for each animal. These averages were then used for statistical comparison between control and treatment groups. Two morphological characteristics, spine neck length and head diameter, were also measured from each reconstructed segment. These data were binned (bin sizes: spine neck length, 0.5 μm; head diameter, 0.1 μm) and then expressed as a frequency distribution with the number of observations in each bin being divided by the total number of observations for the segment. Similar to spine density, frequency distributions were averaged across segments from the NAcC or NAcSh to provide region-specific average frequency distributions for each animal to be used for statistical analysis. Data from each drug treatment group were collected and analyzed in parallel with its corresponding vehicle control group as fluctuations in baseline spine density can occur from cohort to cohort [9].

### Data Analysis

Data were examined for both univariate and multivariate outliers in SPSS (version 20, SPSS, Inc. Chicago, IL). After outliers were removed (*n* = 1 animal per experiment), means were compared using a Student’s *t*-test for spine density analysis. For spine morphology analyses, groups were compared using a two-way ANOVA with main factors of bin and drug treatment, followed by Bonferroni *post-hoc* tests. For all statistical tests, results were considered to be statistically significant if *p* < 0.05.

## Results

**Fig 1:**
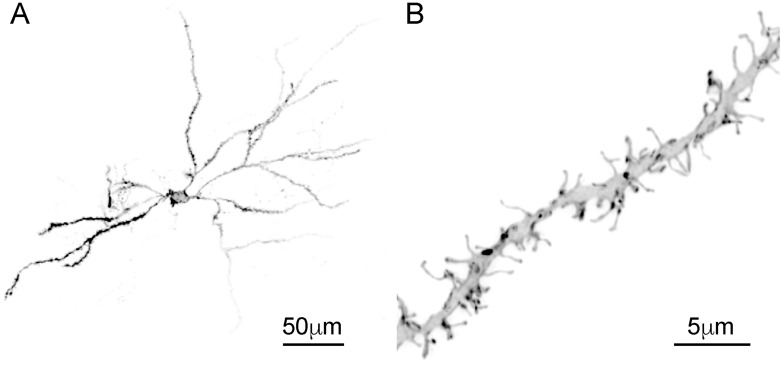
Representative images of DiI-labeled neuron and dendritic segment. (A) Low power magnification of a DiI-labeled medium spiny neuron, *scale bar* 50 μm. (B) High power magnification of a reconstructed dendritic segment from a medium spiny neuron, *scale bar* 5 μm.

**Fig 2:**
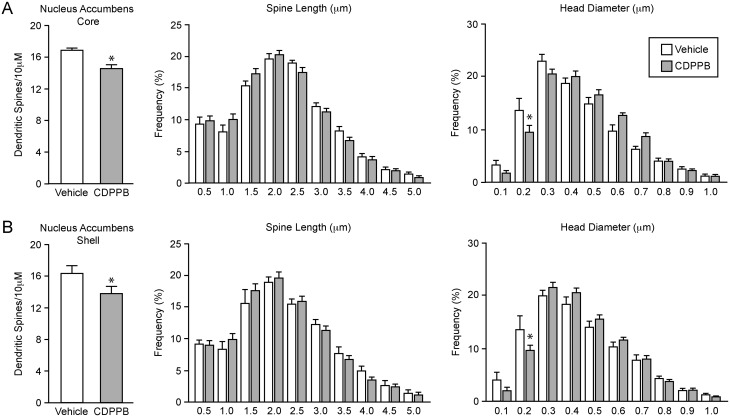
Positive modulation of mGluR5 decreases spine density in the nucleus accumbens but not in the dorsal striatum. (A) Twenty-four hours after systemic administration, CDPPB (5–10 mg/kg, *n* = 18 animals) decreased dendritic spine density and frequency of small head diameter spines (0.2 μm bin) compared to vehicle control (*n* = 8) in the nucleus accumbens core. CDPPB had no effect on neck length. (B) Similarly, CDPPB decreased spine density and frequency of small head diameter spines (0.2 μm bin) in the nucleus accumbens shell, but did not affect neck length. *p < 0.05.

To determine if group I mGluRs can differentially regulate dendritic spine plasticity, we activated either mGluR1a or mGluR5 using two distinct PAMs and analyzed spine density 24 hours later. This time point was chosen based on previous evidence that group I mGluR-dependent signaling is responsible for changes in spine density in the NAc 24 hours after estrogen treatment [9]. For quantification, medium spiny neurons of the NAc and dorsal striatum were ballistically labelled with DiI (Fig 1A) and spines were viewed on individual dendritic segments (Fig 1B). We first examined the effect of mGluR5 activity on spine density by systemically administering (via i.p. injection) the mGluR5 PAM, CDPPB (5 or 10 mg/kg). No differences were observed between the two doses, and consequently the two groups were combined. Twenty-four hours after treatment with CDPPB, spine density in the NAcC was decreased when compared to the vehicle treated group (Fig 2A; *t*(24) = 2.92, *p* < 0.05). CDPPB also decreased dendritic spine density in the NAcSh (Fig 2B; *t*(24) = 2.44, *p* < 0.05) indicating that this effect is not sub-region specific. Importantly, CDPPB administration had no effect on spine density in the dorsal striatum (*t*(24) = 0.408, *p* = 0.69), indicating systemic administration of CDPPB does not necessarily produce the same responses throughout the brain. Two components of spine morphology, spine neck length and spine head diameter, were also measured. CDPPB had no effect on neck length in the NAcC or NAcSh twenty-four hours after administration or on the overall head diameter mean in the NAcC or NAcSh. However, the effect of CDPPB on head diameter in both the NAcC and NAcSh varied by bin, as evidenced by a significant drug x bin interaction (NAcC: *F*(9,216) = 3.12, *p* < 0.05; NAcSh: *F*(9, 216) = 2.15, *p* < 0.05). Specifically, CDPPB treatment reduced the frequency of spines in the 0.2 μm bin in both subregions (NAcC: *t*(24) = 3.45, *p* < 0.05; NAcSh: *t*(24) = 3.24, *p* < 0.05).

**Fig 3:**
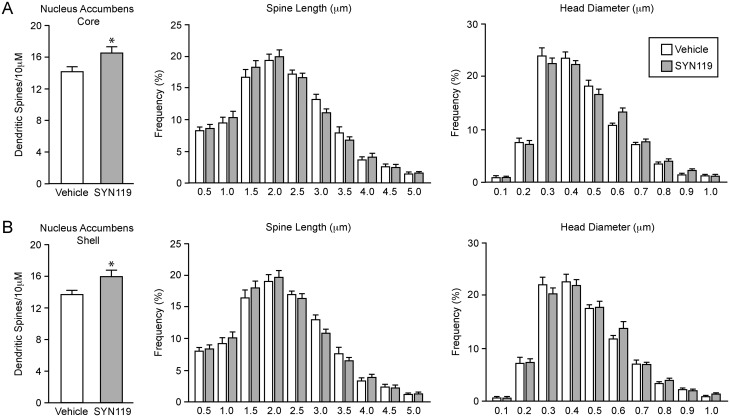
Positive modulation of mGluR1a increases spine density in the nucleus accumbens but not in the dorsal striatum. (A) Twenty-four hours after systemic administration, SYN119 (10 mg/kg, *n* = 10 animals) increased dendritic spine density compared to vehicle control (*n* = 9) in the nucleus accumbens core but had no effect on spine length or head diameter. (B) SYN119 also decreased spine density in the nucleus accumbens shell and did not affect spine length or head diameter. *p < 0.05.

In the next experiment we increased mGluR1a activity with i.p. administration of the mGluR1a PAM, SYN119 (10mg/kg). Twenty-four hours after administration, SYN119 increased spine density in both the NAcC (Fig 3A; *t*(16) = 2.35, *p* < 0.05) and NAcSh (Fig 3B; *t*(17) = 2.17, *p* < 0.05), but did not affect spine neck length or head diameter in either of these regions. Again, mGluR1a modulation had no effect on spine density in the dorsal striatum (*t*(17) = 0.79, *p* = 0.44). Together, these data show that increasing activity of the two group I mGluRs has opposing effects on spine density within the NAc.

**Fig 4:**
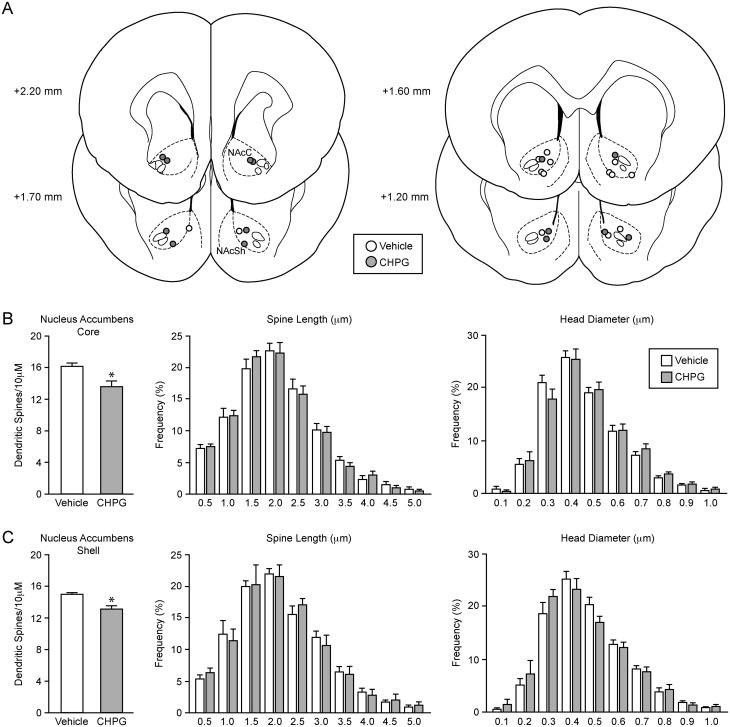
Activation of mGluR5 site-specifically decreases spine density in the nucleus accumbens. (A) Schematic representation of injection sites of either vehicle (open circles) or CHPG (filled circles). Numbers represent distance from bregma, based on the atlas of Paxinos and Watson [16]. (B)Twenty-four hours after local microinfusion to the nucleus accumbens, CHPG (10 μg/side, *n* = 7 animals) decreased dendritic spine density compared to vehicle control (*n* = 7) in the nucleus accumbens core but had no effect on spine length or head diameter. (B) CHPG also decreased spine density in the nucleus accumbens shell and did not affect spine length or head diameter. *p < 0.05.

After observing NAc structural changes following systemic mGluR manipulation, we next determined whether local activation of group I mGluRs was sufficient to produce similar alterations. For this study we chose to focus on the effects of a receptor agonist, rather than a PAM for two reasons. One, direct activation of the mGluR is a more straightforward means to manipulate the system than using an allosteric modulator. Two, while there is one report of PAMs being used via direct injection [15], the solubility requirements of these drugs (i.e. the vehicle in which they are dissolved may affect neuronal function) make their use in direct brain manipulations technically challenging. Hence, we focused on the effects of an mGluR5 agonist as no comparable selective mGluR1a agonist is available. The mGluR5 agonist CHPG (10 μg/0.5 μl/side) or vehicle was bilaterally injected into the NAc and spine density was examined 24 hours later (Fig 4A). CHPG infusion replicated the effects on spine density seen after CDPPB treatment, decreasing spine density in both the NAcC (Fig 4B; *t*(12) = 3.13, *p* < 0.05) and NAcSh (Fig 4C; *t*(11) = 3.98, *p* < 0.05). CHPG did not affect spine neck length or head diameter in either subregion (Fig 4B and 4C).

## Discussion

Group I mGluRs modulate excitatory neurotransmission throughout the brain. One way they affect long-term functioning is by altering dendritic structure. The effects of group I mGluR modulations on spine plasticity have been widely studied in the hippocampus [3–5], yet their impact on structural plasticity in other brain regions has received little attention. Furthermore, indirect evidence suggests that activation of mGluR1a and mGluR5 can have opposite effects on NAc dendritic spine densities [9]. Here we find that systemic activation of the group I mGluRs using compounds that directly modulate these receptors have opposing effects on spine densities. Positive modulation of mGluR5 decreased spine density in both the NAcC and NAcSh and reduced the frequency of small head diameter spines. In contrast, positive modulation of mGluR1a increased spine density in both of these subregions. To study the local effects of mGluR signaling on spine plasticity, we found that direct injection of the specific mGluR5 agonist CHPG into the NAc produced the same spine density changes observed with systemic administration of the mGluR5 PAM. With no suitable selective agonist, we lacked the tools to perform a comparable experiment selectively targeting mGluR1a.

These findings fit into an expanding field of literature defining distinct roles for the two group I mGluRs. The eight mGluR subtypes are grouped by sequence homology, similarity in downstream signal transduction, and receptor pharmacology. The group I mGluRs, mGluR1a and mGluR5, are the subtypes that canonically signal through the activation of Gq G-proteins [1]. Yet, group I mGluR signaling can be highly diverse. Not only is the number of second messenger cascades these receptors can trigger via Gq vast, but group I mGluRs can also couple to other G-protein types as well as activate G-protein independent signaling pathways [17]. Hence, the two group I mGluRs can couple to different subsets of downstream signaling effectors, allowing them to have varied signaling and functions [18,19]. Examples of the group I mGluRs affecting cellular signaling and plasticity in different ways have thus far been found in the striatum [20], hippocampus [18,21], and globus pallidus [22]. Specifically in the nucleus accumbens, mGluR1 and mGluR5 have been shown to have distinct roles in regulating neuronal signaling related to psychostimulant use, with mGluR5 activity reducing excitatory neurotransmission in an endocannabinoid dependent mechanism prior to cocaine experience and mGluR1 activity reducing excitatory neurotransmission in a PKC-dependent mechanism after cocaine exposure [23]. Still, our finding that the two group I mGluRs are having wholly opposite effects on spine plasticity within the same brain region is novel.

For these experiments, we were able to dissect mGluR1a and mGluR5 function by using receptor-specific pharmacological manipulations. Biologically, it would seem that activation of these receptors would coincide in cells where they are co-expressed since they are activated by a common ligand, i.e. glutamate. However, there are multiple biological ways that activation of single mGluR subtypes could be accomplished. In the rat NAc, mGluR1 and mGluR5 only co-localize with each other in approximately half of the number of individual spines [24], leaving the possibility that afferents may often feed into synapses specific to one mGluR subtype. Another possibility is that specific mGluRs are controlled by glutamate-independent means. Research in our lab has explored one example of this mechanism—the regulation of specific mGluR activity through membrane estrogen receptor signaling [9–11,25–29]. As such, in these experiments we utilized ovariectomized female rats to eliminate the effects of ovarian hormones on this signaling system. Estradiol regulation of mGluRs is not the only example of glutamate-independent control of these receptors—cellular prion protein can also act as a ligand to mGluR5 [7]. In both of these examples, signaling molecules other than glutamate are able to specifically activate a single mGluR subtype to initiate a particular line of downstream signaling.

Systemic administration of ligands to regulate specific group I mGluR subtypes is being tested therapeutically. Group I mGluR allosteric modulators in particular have been considered for the treatment of multiple disorders, including drug addiction, where group I mGluRs in the NAc are thought to play an important role. Up until now, the majority of research has focused on decreasing group I mGluR activity with negative allosteric modulators (NAMs), and both mGluR1a and mGluR5 NAMs have been found to decrease drug seeking or drug sensitization [12]. However, recent findings have shown that *positive* modulation of mGluR1a with SYN119 can reduce cue-induced relapse after extended abstinence from cocaine [15], differing from the potentiating effects on drug-seeking seen when mGluR5 is stimulated [30,31]. The differences in these results are likely due in large part to differences in the behavioral models and mGluR interventions being used. However, our results indicate that there are also fundamental differences in the way that mGluR1a or mGluR5 modulators affect NAc plasticity, and these could be contributing to different behavioral effects seen with these drugs. This of course, is only part of the story as the systemic administration of these compounds will have widespread effects upon the nervous system. For example, here we find that increased mGluR5 activity influences NAc spine morphology with a systemic treatment of CDPPB. As local activation in the NAc with CHPG did not affect morphology in a similar way, this effect may be influenced by the activity of CDPPB in other regions of the brain.

Interestingly, just as mGluR1a and mGluR5 stimulation can have divergent effects on neuronal function, regional differences are also observed. Previous research has shown that CDDPB administration has no effect on spine density or morphology in the mPFC [32], and similarly, we find no changes in structure in the caudate with either CDPPB or SYN119. Similarly, estrogen receptors activate mGluR5 signaling in both the female caudate and the NAcC, but structural changes are only observed in the NAc [9,28]. These site-specific modifications may be due to several variables, including intrinsic differences between these brain regions, or differential effects on afferents to these structures after systemic drug application. Lack of a structural change in the caudate is not due to an inability of the cells in this region to respond. In this regard, psychostimulants are well known to produce changes to dendritic spines in both the caudate and NAc [33].

Dendritic spines are highly dynamic structures. Here, we found changes in spine density in the NAc 24 hours after pharmacological manipulation of group I mGluRs, aligning with previous evidence of estradiol and group I mGluR-induced spine changes at this time point in this region [9]. However, it is possible these drugs may have also had transient early effects or more long-term effects on NAc spine density or morphology. Notably, medium spiny neurons exhibit both short and long-term structural changes, often studied with drugs of abuse. For example, single injections of psychostimulants produce acute spine density increases in the NAc, and this form of short-term plasticity is hypothesized to contribute to long-term plasticity and persistent, stable spine changes with multiple exposures to drugs of abuse [33,34]. Additionally, the timing of estradiol-induced changes in dendritic spines across multiple brain regions are both rapid as well as enduring [10,35,36]. Since the goal of this study was to determine whether differential group I mGluR activation could have opposing effects on NAc dendritic spine density or morphology, pursuance of a full time course of mGluR1a- and mGluR5-induced changes in NAc dendritic spine density and morphology is left for future studies.

Our findings—that systemic modulation of individual group I mGluRs differentially regulates spine plasticity in the NAc—highlight the distinct features of mGluR1a and mGluR5 activity. Furthermore, we found that local signaling of mGluR5 is sufficient to bring about structural changes in the NAc, demonstrating that a group I mGluR can be a direct mediator of structural plasticity in this region. These differences make clear that as research continues on group I mGluR plasticity in the NAc and on systemic manipulation of group I mGluRs, it will be important to separately evaluate the activity of mGluR1a and mGluR5 as they are not interchangeable.
